# Preparation and Photocatalytic Properties of Metal-Doped ZnO Nanofilms Grown on Graphene-Coated Flexible Substrates

**DOI:** 10.3390/ma13163589

**Published:** 2020-08-14

**Authors:** Ping Rong, Shuai Ren, Jianchao Jiang, Qi Yu, Liyun Jiang, Yonghong Zhang

**Affiliations:** 1 School of Materials Science and Engineering, Institute of Graphene at Shaanxi Key Laboratory of Catalysis, Shaanxi University of Technology, Hanzhong 723001, China; rongping1994@163.com (P.R.); renshuai2006@163.com (S.R.); jiangjcsxlg@163.com (J.J.); zhangyhor@163.com (Y.Z.); 2 School of Physics and Telecommunication Engineering, Shaanxi University of Technology, Hanzhong 723001, China; lyjiang2014@126.com

**Keywords:** ZnO, photocatalytic, doped, graphene, nanofilms

## Abstract

A series of metal element (Al, Fe, Mg and Ni)-doped ZnO (M-ZnO) photocatalysts have been successfully synthesized on graphene-coated polyethylene terephthalate (GPET) flexible substrate via the hydrothermal method. The effects of doped metals in ZnO were also studied on the crystal structure, morphology and photocatalytic performance. The photocatalytic experiment results indicated that, compared with Al-, Mg- and Fe-ZnO/GPET photocatalysts under ultraviolet (UV) light irradiation, Ni-ZnO/GPET had better photocatalytic activity, and the degradation rate of methylene blue (MB) was 81.17%. Meanwhile, the mechanism of enhancing the photocatalytic activity of metal element-doped ZnO is also discussed. It is concluded that, after doping with metal elements, electrons and holes are prevented from recombination by trapping electrons of the ZnO/GPET conductive band, thereby improving the photocatalytic activity.

## 1. Introduction

In recent years, water pollution has become one of the most serious problems endangering human health, the environment and social sustainable development. Semiconductor photocatalysis is a green environmental protection technology with the ability to remove organic pollutants in the air and water, and it is an effective way to solve the energy and environmental crisis [1]. At present, metal oxides such as TiO_2_ [2], SnO_2_ [3] and ZnO [4] have been used by researchers around the world to remove various pollutants by photocatalytic methods. Among them, ZnO exhibits excellent photocatalytic activity under ultraviolet (UV) light irradiation, and its high exciton binding energy (60 meV) provides it with efficient exciton emission at room temperature, making it an attractive wide band gap (3.37 eV) semiconductor photocatalyst [5,6]. In addition, ZnO as a photocatalyst also has the advantages of low cost, non-toxicity and high absorption of light quantum. However, the rapid recombination of photogenerated carriers suppresses the photocatalytic efficiency of ZnO. Studies have found that element doping is an effective method to improve the photocatalytic performance of ZnO by inhibiting the recombination of electron-hole pairs [7]. Traditional powder catalysts have problems such as difficult solid–liquid separation, difficult recycling and practical application [8]. To overcome these problems, thin-film photocatalysts can be used. ZnO films with photocatalytic properties were prepared on polyetherimide flexible substrates using spray pyrolysis deposition by Ameur et al. [9]. Sanchez et al. successfully fabricated ZnO nanorod (NR) film polyethylene terephthalate (PET) substrate and studied its photocatalyst performance [10]. There are many types of flexible substrates, including conductive polymer films, graphene, metal foil, carbon fiber cloth and so on. Among them, graphene, as a two-dimensional carbon nanomaterial with zero band gap, has high specific surface area (~2600 m^2^g^−1^), high carrier mobility (15,000 cm^2^V^−1^s^−1^), high conductivity and transparency, so it is considered to be an excellent flexible growing substrate [11]. In addition, the combination of graphene and semiconductor materials can realize electron conduction between the two and suppress the recombination efficiency of photo-generated carriers [12,13,14]. Meanwhile, most studies have shown that doping metal ions can be devoted to enhancing the photocatalytic performance of ZnO structures [15,16,17]. However, to the best of our knowledge, there are few reports about metal-doped ZnO prepared on graphene-coated PET (GPET) by the hydrothermal method and applied in the photocatalytic field.

In this paper, M-ZnO (M = Al, Fe, Mg and Ni) composites were synthesized by a simple and green hydrothermal method by using GPET as the growth substrate. The prepared samples were fundamentally characterized. The photocatalytic performance of ZnO/GPET composite structures doped with different elements was analyzed, and the possible photocatalytic mechanisms of M-ZnO/GPET were investigated.

## 2. Experimental Methods

### 2.1. Synthesis of M-ZnO Nanofilms on GPET Substrates

The M-ZnO nanofilms were successfully synthesized on GPET substrates. The substrates were purchased from 2D Carbon Tech Inc., LTD (Changzhou, China). After ultrasonically cleaning the flexible substrate of GPET with acetone (10 min), methanol (10 min) and deionized (DI) water, the ZnO seed layers with a thickness of 30 nm were sputtered on the surface of GPET substrates by radio frequency magnetron sputtering to control the growth direction of ZnO. We weighed a certain amount of zinc nitrate hexahydrate (Zn(NO_3_)_2_·6H_2_O) and hexamethylenetetramine (C_6_H_12_N_4_) in 30 mL of DI water, added 0.1 mol/L aluminum (Al_2_O_3_) (or iron nitrate (Fe(NO_3_)_3_·9H_2_O), magnesium nitrate hexahydrate (Mg(NO_3_)_2_·6H_2_O) and nickel nitrate hexahydrate (Ni(NO_3_)_2_·6H_2_O)) as a dopant, and stirred the mixture under weak magnetic conditions for 30 min to completely dissolve to form growth solution. 

The configured growth solutions were transferred to Teflon-lined stainless-steel autoclaves, and the GPET substrates sputtered with the ZnO seed layer were vertically immersed in the solution. After this, the autoclaves were sealed and put into an oven and heated at 95 °C for 6 h. The products were washed with DI water and dried naturally at room temperature.

### 2.2. Structural Characteristics

X-ray diffraction (XRD, by Rigaku D/MAX-Ultima with Cu Kα radiation, Rigaku, Tokyo, Japan) was used to characterize the crystal structure of samples, and a field emission scanning electron microscope (FESEM, by FEI Magellan 400, Milpitas, CA, USA) was used to observe the micro-morphology. X-ray photoelectron spectroscopy (XPS, by ESCALAB Xi+, ThermoFisher, Dartford, UK) was mainly used to characterize the surface elements of M-ZnO/GPET and valence state. The Cary Eclipse fluorescence spectrophotometer (wavelength 325 nm) from Agilent (Santa Clara, CA, USA), was used to measure the photoluminescence (PL) spectrum of the sample to detect its photoluminescence characteristics.

### 2.3. Photocatalytic Measurement

**Figure 1:**
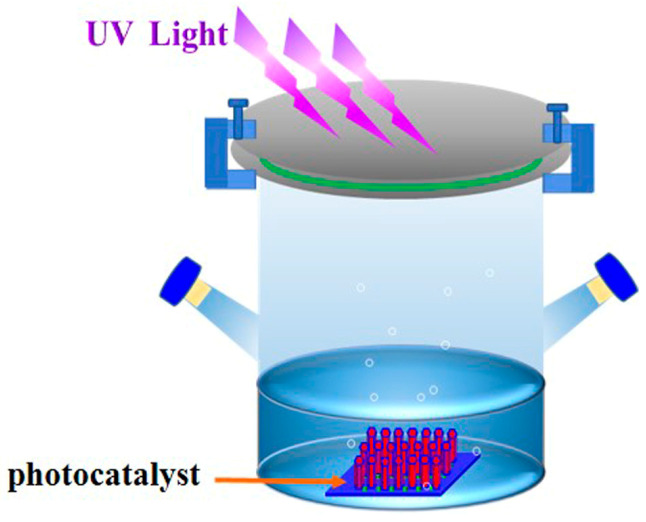
Diagram of simple photocatalytic experimental apparatus.

The photocatalytic performance of M-ZnO/GPET nanofilms was analyzed by measuring the degradation rate of methyl blue (MB) solution under UV irradiation, and Figure 1 is a diagram of a simple experimental device, configured with 3 mg/L MB solution and added to the photocatalytic reactor. After rinsing the ZnO film with a small amount of MB solution, it was immersed in the reactor for dark reaction to enable the MB solution to reach the adsorption–desorption equilibrium. Then, it was placed under a 500 W Xe lamp (CEL-S500) for photocatalytic reaction by UV irradiation. The MB solution was quantitatively measured every 30 min and the absorbance was measured by a UV spectrophotometer (λ max = 664 nm).

## 3. Results and Discussion

### 3.1. Morphology and Structure of M-ZnO/GPET Composites

**Figure 2:**
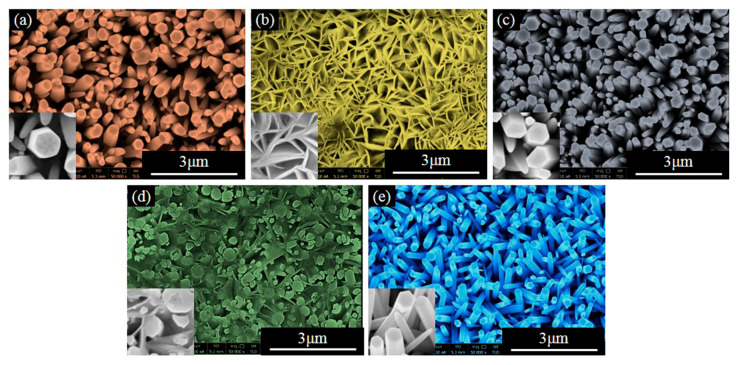
FE-SEM images of (**a**) ZnO/GPET, (**b**) Al-ZnO/GPET, (**c**) Mg-ZnO/GPET, (**d**) Ni-ZnO/GPET and (**e**) Fe-ZnO/GPET.

FE-SEM images were taken in order to analyze the microscopic morphology of M-ZnO/GPET samples and the results can be seen in Figure 2a–e. As shown in Figure 2a,c, in the case of the undoped and doped Mg elements, ZnO was a vertically arranged, different size of the NR’s structure, suggesting that undoped and Mg-ZnO/GPET have a good degree of orientation and Mg doping does not significantly change the morphology of the ZnO. With the addition of Ni or Al elements, the structure of ZnO is changed from NR to nanosphere (NS). It is not difficult to see that Ni-ZnO/GPET has an NR/NS composite structure (Figure 2d). Al-ZnO/GPET NSs, with smooth surfaces, still grow perpendicular to the GPET substrate and are connected to form a network structure, which can be clearly observed in Figure 2b. This indicates that the doping of Al or Ni could affect the growth mechanism of ZnO nanostructures. Figure 2e shows that Fe-ZnO nanomaterials are regular hexagonal NRs that uniformly and densely cover the surface of the GPET substrate.

**Figure 3:**
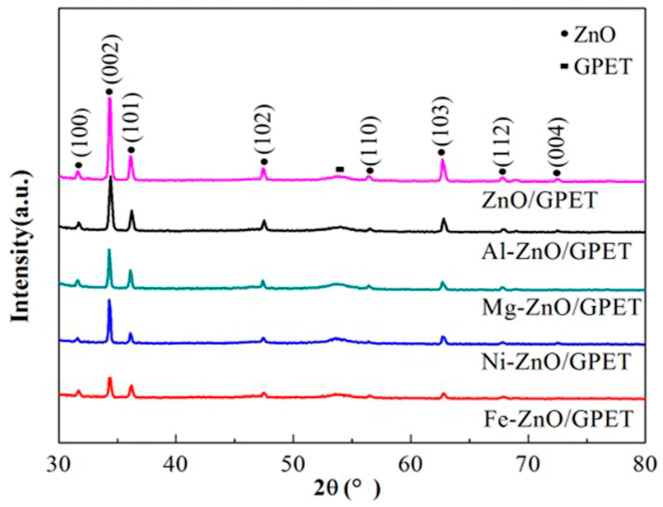
XRD patterns of ZnO/GPET and M-ZnO/GPET nanomaterials.

The crystal structure and phase purity of pure ZnO/GPET and M-ZnO/GPET samples were characterized by XRD. As shown in Figure 3, the diffraction peaks at 31.6°, 34.3°, 36.1°, 47.4°, 56.6°, 62.7°, 67.8°, 72.4° correspond to (100), (002), (101), (102), (110), (103), (112) and (004) crystal planes of ZnO, respectively, which is basically consistent with the standard ZnO pattern (JCPDS No. 36-1451). It is indicated that the ZnO samples before and after doping are all single-phase hexagonal wurtzite structures. Except for the peak near 55°, which is the diffraction peak of the GPET flexible substrate, other peaks are attributed to the ZnO characteristic peak, and no impurity peaks appear. This proves that the doping elements, namely Al, Fe, Ni and Mg, all substitute Zn^2+^ into the ZnO lattice in the form of ions. It is worth mentioning that, in the obtained wurtzite-type ZnO crystals, with the exception of Fe-ZnO/GPET, the diffraction peak intensity of the (002) crystal plane of other samples is significantly higher than other crystal planes. This indicates that the sample has a high c-axis preferential orientation, which is consistent with the FE-SEM results. After doping Al, Mg, Ni and Fe elements, the intensity of the XRD diffraction peak of M-ZnO/GPET changed, indicating that metal ion doping may affect the crystallinity of ZnO.

**Figure 4:**
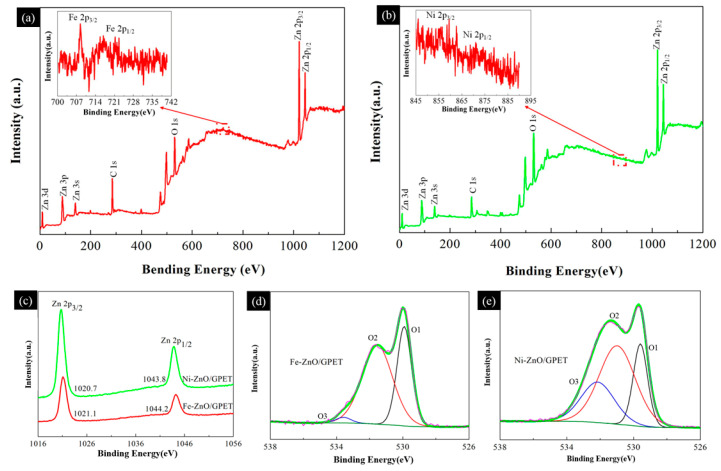
XPS spectra (**a**) Fe-ZnO/GPET, (**b**) Ni-ZnO/GPET, (**c**) Zn 2p, (**d**) O 1s of Fe-ZnO/GPET and (**e**) O 1s of Ni-ZnO/GPET. The inset corresponds to the photoelectron peak of the doping element.

In order to obtain information on the surface composition and valence state of the elements, XPS research was carried out on Fe-ZnO/GPET and Ni-ZnO/GPET nanocomposites (as shown in Figure 4). As can be seen from Figure 4a,b, the full XPS spectra of Fe-ZnO/GPET and Ni-ZnO/GPET have three types of strong electron peaks corresponding to Zn, C and O elements, respectively. Among them, C 1s presents a single strong peak near 284.6 eV, which may be attributed to sp^2^ hybrid carbon atoms [18]. The two optoelectronic peaks of Fe 2p are shown in the inset in Figure 4a, respectively, which are Fe 2p_1/2_ at high binding energy (721.3 eV) and Fe 2p_3/2_ at low binding energy (708.4 eV). The spin orbit is separated to 12.9 eV, which means that Fe exists in an oxidation state of Fe^3+^ [19,20,21]. The typical XPS peaks of Ni 2p_3/2_ and Ni 2p_1/2_ are 855.7 eV and 871 eV, respectively (Figure 4b), which are consistent with the reported values of Ni 2p [22]. From the high-resolution Ni 2p spectrum, the photoelectron peaks of Ni 2p_3/2_ and Ni 2p_1/2_ are not obvious, yet the peak position is consistent with the literature, indicating that Ni exists in an oxidation state of Ni^2+^. The intensity of the Ni 2p photoelectron peak is weak, probably because the content of the Ni element actually incorporated into the ZnO crystal is relatively small.

The XPS spectra of O1s shown in Figure 4d,e are asymmetric, indicating the presence of multi-component oxygen on its surface. The peak (O1) at low binding energy is attributed to the Zn-O bond [23]. The peak of intermediate level (O2) is related to O_2_^−^ ions in the oxygen deficient regions within the matrix of ZnO, and the high energy peak (O3) is related to the presence of loosely bound oxygen forms, such as CO_3_, adsorbed O_2_ or H_2_O, adsorbed on the surface of ZnO [23,24]. Figure 4c shows the combined energy of Zn 2p, with peaks around 1021 eV and 1044 eV belonging to Zn 2p_2/3_ and Zn 2p_1/2_, respectively, suggesting that Zn exists in a 2+ chemical state in the samples. In addition, the distance between the two peaks is 23 eV, which is consistent with the energy splitting of ZnO [25].

### 3.2. Photocatalytic Performance

**Figure 5:**
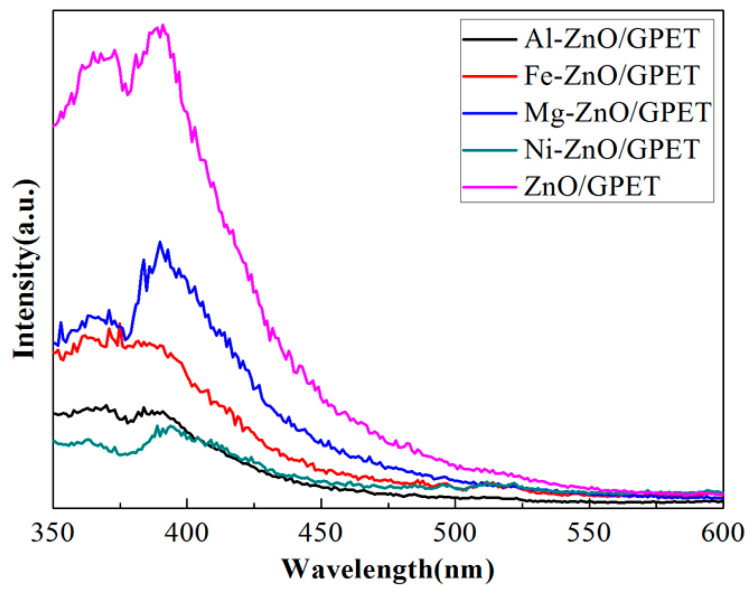
Photoluminescence (PL) spectra of all M-ZnO/GPET composites.

Figure 5 is the PL emission spectrum of M-ZnO/GPET nanomaterials. It can be seen that all samples have a near-UV emission peak at around 389 nm, which is mainly due to the near-band edge transition of electrons. Pure ZnO/GPET has the highest PL emission intensity in all films, which indicates that its electron-hole pair recombination efficiency is high. Compared with undoped ZnO/GPET nanomaterials, the UV luminescence peak of M-ZnO/GPET is significantly weakened, which indicates that metal doping can effectively reduce the recombination efficiency of electron-hole pairs and is more conducive to the improvement of photocatalytic activity [26]. However, in this study, the photocatalytic activity of Fe-ZnO/GPET is better than that of Al-ZnO/GPET. This abnormal PL behavior has also been reported by other researchers, possibly due to the lower crystallinity of Fe-ZnO [27].

**Figure 6:**
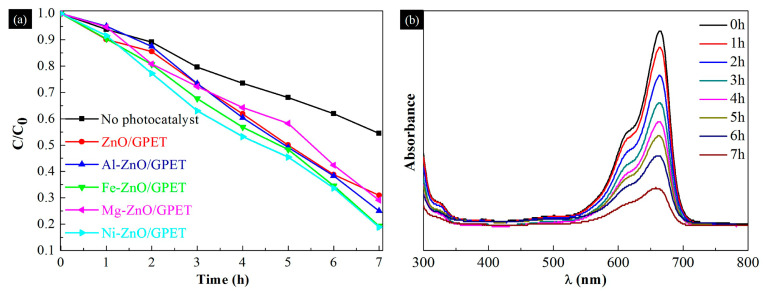
(**a**) Photo-degradation rate of methylene blue (MB) stimulated by UV in the presence of M-ZnO/GPET samples. (**b**) Temporal evolution of the UV-vis absorption spectra corresponding to MB in the presence of Ni-ZnO/GPET.

The MB solution was degraded by UV irradiation under a xenon lamp, and the effect of metal elements doping on the photocatalytic performance of ZnO/GPET nanocomposites was studied. Figure 6a is the degradation rate graph of ZnO/GPET and M-ZnO/GPET as photocatalysts after being irradiated with a xenon lamp for 7 h. For comparison, the photocatalytic degradation efficiency of MB was analyzed without adding a photocatalyst. The results showed that the efficiency of MB degradation is only 45.57% without a photocatalyst. It can be seen that the addition of ZnO photocatalysts has a certain effect on the degradation of MB. Under light irradiation, the photocatalytic efficiency values of ZnO/GPET, Mg-ZnO/GPET and Al-ZnO/GPET were lower, which were 69.08%, 70.86% and 75%, respectively. In contrast, Fe and Ni single-doped ZnO/GPET nanostructures show better photocatalytic efficiency in MB solution degradation, of which Ni-ZnO/GPET has the highest photocatalytic efficiency, followed by Fe-ZnO/GPET (80.69%). Figure 6b is the absorption spectrum of the MB solution under the photocatalysis action of the Ni-ZnO thin film, and the strong absorption band at 664 nm is the maximum wavelength of the MB solution. With the increase in the illumination time, the intensity of the characteristic absorption peak gradually decreases at a wavelength of 664 nm. After 7 h, Ni-ZnO/GPET nanoclusters can degrade 81.17% MB dye. This may be due to the incorporation of Ni atoms into the ZnO lattice, which changes the morphology and structure of ZnO, evolving from NRs to dense NR/NS composite structures. Ni-ZnO NRs/NSs have a larger specific surface area, which provides more adsorption points for MB, thus increasing its adsorption capacity [28]. Heinonen et al. studied the photocatalytic performance of ZnO films prepared on stainless-steel substrates, and the results showed that the optimal sample can degrade 82% of MB solution under 7 h of UV light irradiation [29]. Although its photocatalytic activity is 0.83% higher than that of Ni-ZnO/GPET, the size of the photocatalyst sample is 7.5 cm × 2.5 cm, while the size of all photocatalysts in our research is 1 cm × 2 cm.

Based on the above experimental results, a possible photocatalytic mechanism is proposed. Firstly, because the band gap of doped metals is relatively narrow, the impurity level between the conduction band (CB) and the valence band (VB) of ZnO is formed. When the photocatalyst is irradiated with UV light, the photon energy is higher than or equal to the band gap of M-ZnO/GPET, so that electrons are excited from the VB to the CB and create a positively charged hole. On one hand, doped metal ions hinder electron-hole recombination by trapping electrons in the CB of ZnO/GPET and produce more superoxide radical anions (O_2_^−^). On the other hand, the positive holes left in the VB can react with H_2_O to form highly active hydroxyl groups (•OH) [30]. •OH and O_2_^−^ react with the MB adsorbed on the ZnO/GPET nanophotocatalyst to produce H_2_O and CO_2_, which leads to degradation and decolorization [31]. The reaction formula during degradation is as follows:

ZnO + Light (hv) → ZnO (e^−^ + h^+^) (1)

ZnO (e^−^) + M → ZnO−M (e^−^) (2)

ZnO−M (e^−^) + O_2_ → •O_2_^−^ (3)

•O_2_^−^ + H^+^ → HO_2_• (4)

2HO_2_• → H_2_O_2_ + O_2_ (5)

H_2_O_2_ + e^−^ →•OH + OH^−^ (6)

OH& •O_2_^−^ + MB → CO_2_ + H_2_O (7)

## 4. Discussion

In summary, M-doped ZnO nanomaterials were successfully fabricated on GPET substrate by the hydrothermal method, and their photocatalytic properties were studied. In comparison to Al-, Fe-, Ni- and Mg-ZnO/GPET, the as-prepared Ni-ZnO/GPET exhibits better photocatalytic performance, and its degradation rate is 12.09% higher than that of pure ZnO/GPET. In addition, the doped metal elements can capture the electrons in CB of ZnO/GPET and effectively separate the photogenerated electron-hole pairs, which has the benefit of improving the photocatalytic performance. Considering the hydrothermal method, with its advantages of simple preparation and low cost, Ni-ZnO/GPET nanomaterials, as the most efficient catalyst, have potential application value in solving the problem of water pollution caused by non-biodegradable organic dyes and wastewater.
